# Identification and molecular characterization of the first complete genome sequence of Human Parechovirus type 15

**DOI:** 10.1038/s41598-020-63467-w

**Published:** 2020-04-21

**Authors:** Maria Dolores Fernandez-Garcia, Etienne Simon-Loriere, Ousmane Kebe, A. Sakuntabhai, Kader Ndiaye

**Affiliations:** 1 Institut Pasteur, Dakar, Senegal; 2 Institut Pasteur Paris, Paris, France

**Keywords:** Phylogenetics, Next-generation sequencing, Virology

## Abstract

Using a metagenomics approach, we have determined the first full-length genome sequence of a human parechovirus type 15 (HPeV15) strain, isolated from a child with acute flaccid paralysis and co-infected with EV-A71. HPeV15 is a rarely reported type. To date, no full-length genome sequence of HPeV15 is available in the GenBank database, where only limited VP1 sequences of this virus are available. Pairwise comparisons of the complete VP1 nucleotide and deduced amino acid sequences revealed that the study strain belongs to type 15 as it displayed 79.6% nucleotide and 93.4% amino acid identity with the HPeV15 prototype strain. Comparative analysis of available genomic regions and phylogenetic analysis using the P2 and P3 coding regions revealed low nucleotide identity to HPeV reference genomes. Phylogenetic and similarity plot analyses showed that genomic recombination events might have occurred in the UTRs and nonstructural region during HPeV15 evolution. The study strain has high similarity features with different variants of HPeV3 suggesting intertypic recombination. Our data contributes to the scarce data available on HPeVs in Africa and provides valuable information for future studies that aim to understand the evolutionary history, molecular epidemiology or biological and pathogenic properties of HPeV15.

## Introduction

Human parechoviruses (HPeVs) are non-enveloped viruses that belong to one of the four species of genus *Parechovirus* within the *Picornaviridae* family. HPeV positive-sense single-stranded RNA genome is about 7300 bases in length, flanked by 5′ and 3′ untranslated regions (UTRs). The genome encodes a polyprotein which is cleaved by the viral protease (3 C) to produce the mature structural (VP0, VP3 and VP1) and nonstructural (2A-C, 3A-D) proteins^1^. HPeVs can be transmitted through the fecal-oral and respiratory routes^1,2^. HPeV infections are common throughout the world and most often cause asymptomatic or mild gastrointestinal or respiratory symptoms^1,2^. However, syndromes such as encephalitis, aseptic meningitis, acute flaccid paralysis (AFP), sepsis and myocarditis have been increasingly recognized in primary HPeV infection in neonates and infants^2^. After EVs, HPeVs are considered the second most common cause of central nervous system (CNS) viral infections^2^. However, HPeV infections are currently underdiagnosed given that routine diagnostic assays are generally lacking in clinical settings^1,3^. Following HPeV intestinal replication, infectious viruses are shed in feces where viruses can be detected by PCR and virus isolation^3^. However, HPeVs are known for poor adaptation to cell cultivation systems and not all types replicate in cell lines routinely used for enterovirus (EV) detection^4,5^. Because specific types could be missed or underdiagnosed using viral isolation, molecular identification methods are highly recommended for the routine detection of HPeVs in clinical samples^3,4^. To date, 19 different HPeV types (HPeV1 to 19) have been described based on sequence similarity within their VP1 capsid protein (www.picornaviridae.com). HPeV1 and 2 (originally known as EVs echo22 and echo23, respectively) were first isolated in the 1950’s and reclassified in 1999 as members of a separate new genus, *Parechovirus*^6^. Due to difficulties of HPeVs detection in cell culture, it was not until 2004 when further types started to be identified (reviewed in^1^). The spectrum of clinical manifestations and epidemiology of HPeV infections differ among virus types. The most common types are HPeV types 1 to 8, and HPeV types 1, 3 and 6 account for the majority of infections worldwide^1,2^. Of all the HPeV types, HPeV3 is the most frequently involved in severe disease including neurological illness in infants^1,7,8^. Epidemiological and clinical data related to types 7–19 is scarce due to the low number of cases reported globally. The HPeV15 prototype strain BAN-11614 was first reported from Bangladesh from a non-human primate sampled between 2007 and 2008^9^. Since then, it was detected in children from Pakistan in 2008–2010 (seven cases with acute dehydrating gastroenteritis)^4,10^ and from Ghana in 2007–2008 (one case with diarrhea and two healthy controls)^11^. However, to date, only VP1 sequences of HPeV15 were available in GenBank, which were used in the present study to perform evolutionary analysis of HPeV15: one from Bangladesh^9^ (JX219573), six from Pakistan^4^ (KF626452-53, KF626458-61) and three from Ghana^11^ (KY931649-51). The sequence of the HPeV15 strain (NIHPAK-RGH2476) from Pakistan, detected in a child with acute gastroenteritis in 2009–2010, is not available in GenBank^10^. Prior to this study, complete genome sequences of HPeVs were only available for 13 out the 19 types described (HPeV 1–8, 14, 16–19). The majority (>70%) of complete genomes are from HPeV types 1 and 3. Clearly, increased access to molecular detection and sequencing methods are needed to expand the number of complete HPeV genomes. This will help future studies to further the understanding of the origin, evolution, spread and relationship between the biology/pathogenesis of HPeVs. In this study, we present the identification and analysis of the first complete genome sequence of a HPeV15 strain (hereafter referred to as NIG13194).

## Results

### Sample collection

The study strain HPeV15 NIG13194 was isolated in 2013 from a stool sample from a child with AFP living in the district of Mirriah, in the Zinder region of Niger. The stool sample was collected in the framework of AFP surveillance activities in support of the global polio eradication initiative and sent to the WHO Reference Intercountry Laboratory for poliomyelitis surveillance in Institut Pasteur of Dakar (Senegal). Retrospective analysis of non-polio enterovirus (NPEV) isolates obtained through routine poliomyelitis surveillance activities by partial VP1 typing revealed isolate NIG13194 as an EV-A71^12^. Subsequent metagenomic Next Generation Sequencing (NGS) of isolate NIG13194 revealed co-infection with HPeV15^13^. The child was a 1-year-old boy with AFP. Flaccid paralysis symptoms appeared on February 26^th^, 2013 and sample collection was done on March 6^th^, 2013 when the patient still presented paralysis.

### Full-length genomic characterization and analysis

The nucleotide (nt) sequence of NIG13194 was generated with an average coverage of 78X. De novo assembly and iterative mapping produced a genome scaffold covered by 15272 reads (0.04% of the total assembled reads).

The full-length sequence of NIG13194 consists of 7,298 nt in length excluding the 3′-poly (A) tail. The overall base composition of the study strain genome was 32.5% A, 20.4% G, 19.2% C and 27.9% U. NIG13194 contained a 5′untranslated region (UTR) of 680 nt, a single ORF of 6531 nt encoding a putative polyprotein precursor of 2177 aa, and a 3′UTR of 99 nt preceding the poly(a) tract. The polyprotein of NIG13194 comprised capsid proteins VP0 (289 aa), VP3 (256 aa), and VP1 (226 aa), and nonstructural proteins 2 A (149 aa), 2B (122 aa), 2 C (329 aa), 3 A (117 aa), 3B (20 aa), 3 C (200 aa), and 3D (469 aa). The NIG13194 strain was further characterized by comparing the nt sequence and deduced amino acid (aa) sequences in different regions of the genome with those of HPeV prototype strains available in the GenBank database (Table 1). In order to identify the type of the HPeV detected, the VP1 sequence of strain NIG13194 was compared with those of 19 HPeV reference strains. Based on the standard criteria for classification of parechoviruses (nt and aa sequence identity of VP1 ≥ 77% and ≥87%, respectively^14^), strain NIG13194 was confirmed to be a HPeV15 as its complete VP1 coding sequence displayed 79.6% nt and 93.4% aa similarity with the HPeV15 prototype strain BAN-11614 (Table 1). Type HPeV15 was also confirmed with the RIVM typing tool (http://www.rivm.nl/mpf/enterovirus/typingtool/). Following alignment of the VP1 coding region of NIG13194 with HPeV15 prototype strain BAN-11614, nine deletions were observed at the 3′end of the VP1 (nt 649–651 and nt 666–671, numbered according to strain BAN-11614). Comparison of the complete ORFs of NIG13194 with other prototypes showed low nt identities (75.5 to 79.3%) and low aa identities (71.2 to 90.6%). The closest related HPeVs were HPeVs -3, -7 and -18 with nt identity of 79.3% and aa identity of 74.2%-90.6% (Table 1).

**Table 1:** Nucleotide sequence and deduced amino acid sequence identities between the study strain NIG13194 and representative HPeV strains for each type.

		Genomic region													
		5′UTR	VP0	VP3	VP1	2 A	2B	2 C	3 A	3B	3 C	3D	3′UTR	ORF	Ref
% Nucleotide (amino acid) identity	HPeV1*	83.9	70.2 (73.1)	68.3 (78.9)	67.5 (74)	75.8 (89.3)	80.3 (96.7)	79 (92.4)	77.5 (64.9)	75 (95)	82 (98)	83.2 (96.3)	75.5	76.4 (87.6)	^64^
	HPeV2*	83	69 (74.4)	70 (78.5)	67.5 (75.2)	74.7 (86)	76.7 (95.9)	75.9 (86.3)	76.9 (62.3)	85 (95)	79.5 (98)	83.3 (95.3)	82.9	75.5 (86.2)	^65^
	HPeV3*	83.2	71.8 (80.6)	72.1 (82.4)	75.3 (80)	75.3 (86.7)	86.6 (100)	85.8 (98.1)	83.5 (75.2)	68.3 (90)	79.6 (97.5)	83.9 (95.7)	84	79.3 (90.6)	^45^
	HPeV4*	86.3	70.3 (72)	69.9 (82)	68.4 (73.2)	76.6 (88)	81.9 (100)	85.3 (97.8)	88.6 (78.6)	78.3 (100)	78 (97.5)	84.7 (96.5)	82.9	78.3 (89.1)	^31^
	HPeV5*	84.3	70.5 (73.4)	68.7 (76.4)	64.2 (70.6)	78 (89.3)	81.9 (98.3)	80.4 (95.4)	78.6 (68.3)	71.6 (85)	81.5 (98.5)	85 (96.1)	81.9	76.9 (71.9)	^66^
	HPeV6*	85.1	69.7 (72.4)	69.2 (76.9)	66.4 (67.9)	76.6 (86.7)	78.6 (97.5)	79.3 (91.4)	78.1 (65.8)	71.6 (85)	81.6 (99)	83.7 (95.7)	81.9	76.4 (86.2)	^44^
	HPeV7*	87	67.9 (70.9)	72.9 (81.6)	70.2 (77.8)	77.7 (86)	86.3 (99.1)	87.8 (97.2)	88.3 (81.1)	78.3 (95)	80.8 (98.5)	83.8 (95.3)	81.9	79.3 (72.5)	^36^
	HPeV8*	82	69.5 (73.7)	69.2 (76.5)	71.2 (74.1)	76.2 (87.3)	78.9 (98.3)	79.3 (92.4)	75.2 (62.3)	78.3 (95)	82.1 (98)	83 (96.3)	87.2	76.6 (87.2)	^67^
	HPeV9*	—	—	—	69 (74)	—	—	—	—	—	—	—	—	—	^21^
	HPeV10*	—	—	—	71.8 (79.2)	—	—	—	—	—	—	—	—	—	^21^
	HPeV11*	—	—	—	73.6 (84.9)	—	—	—	—	—	—	—	—	—	^21^
	HPeV12*	—	—	—	71.7 (75.6)	—	—	—	—	—	—	—	—	—	^21^
	HPeV13*	—	—	—	70.5 (71.2)	—	—	—	—	—	—	—	—	—	^21^
	HPeV14	90.7	73.2 (77.8)	72.6 (82)	69.7 (77.8)	77.4 (89.9)	78.6 (98.3)	79.1 (91.4)	75.2 (89.7)	73.3 (85)	81.6 (98.5)	83.5 (96.1)	86	77.3 (88.9)	^68^
	HPeV15*	—	—	—	79.6 (93.4)	—	—	—	—	—	—	—	—	—	^9^
	HPeV16	78.5	69.3 (74.8)	69.9 (78.1)	69.9 (71.6)	75.8 (84.5)	82.7 (99.1)	78.3 (91.1)	78.6 (92.3)	76.6 (90)	83.5 (99.5)	85.7 (96.8)	83.8	77.3 (87.3)	^26^
	HPeV17*	86.4	74 (70.2)	74 (84.3)	70.6 (74.8)	74.6 (87.3)	81.1 (95.9)	78 (91.1)	76.7 (70.9)	81.6 (90)	82 (98.5)	86.3 (96.8)	86.1	78.5 (73.5)	^24^
	HPeV18	84.4	70.4 (81.3)	72.1 (82.4)	73.3 (78.7)	76.2 (87.3)	84.9 (99.1)	85.4 (98.4)	88.6 (80.3)	80 (85)	79.5 (98.5)	84 (96.5)	67	79.3 (74.2)	^38^
	HPeV19	60.1	69.5 (70.5)	70.8 (79.3)	70.8 (77.8)	77.3 (86)	78.4 (98.3)	82 (96)	84.3 (76.9)	78.3 (100)	80 (99)	82.9 (94.6)	35.1	77.6 (71.2)	^38^

Sequences for the HPeV strains, HPeV1 (Harris L02971), HPeV2 (Williamson, AJ005695), HPeV3 (A308/99, AB084913), HPeV4 (K251176-02, DQ315670), HPeV5 (CT86-6760, AF055846), HPeV6 (NII561, AB252582), HPeV7 (PAK5045, EU556224), HPeV8 (BR/217, EU716175), HPeV9 (BAN2004-10902, JX219575), HPeV10 (BAN2004-10903, JX219568), HPeV11 (BAN2004-10905, JX219574), HPeV12 (BAN2004-10904, JX219567), HPeV13 (BAN2005-10901, JX219579), HPeV14 (V3C, MG571809), HPeV15 (BAN-11614, JX219573), HPeV16 (CMRHP2/CMR/2014, MH933779), HPeV17 (M36/CI, KT319121), HPeV18 (11Chzj207, KT879915), HPeV19 (67Chzj11, KT879920). – indicate no sequencing data available; * indicate prototype strains.

### Phylogenetic analysis

**Figure 1:**
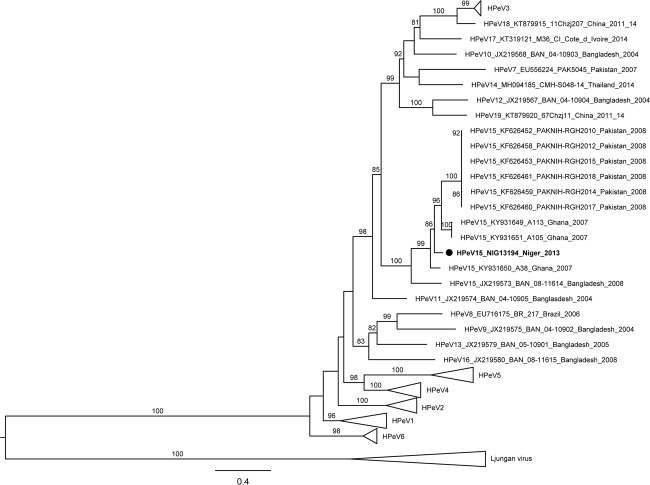
Phylogenetic tree of partial VP1 (660 bp in length) including the study strain, global HPeV15 strains and representative global HPeV strains from the GenBank database. The black circle indicates HPeV15 isolate in this study. The maximum likelihood tree was constructed using IQ-TREE using the GTR + F + I + G4 substitution model, with ultrafast bootstrap. Bootstrap values above 80% are indicated in branch nodes. Scale bars indicate nucleotide substitutions per site. The name of each strain includes type designation, GenBank accession number, strain name, country of origin, and year of detection.

**Figure 2:**
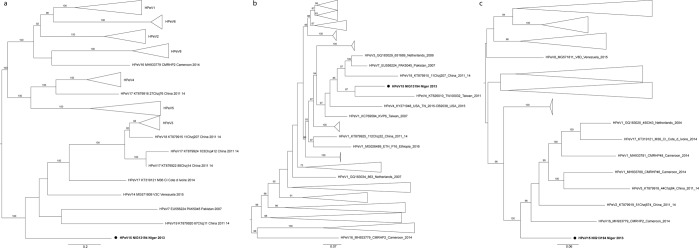
Phylogenetic relationships based on the P1, P2 and P3 coding sequences of the NIG13194 study strain and other 133 fully sequenced HPeV global strains. The phylogenetic trees were inferred from the nucleotide sequence alignment of a ~ 2313, 1800 and 2418 bp-sized nucleotide sequence data within the P1 (**a**), P2 (**b**), and P3 (**c**) coding regions, respectively, using IQ-TREE. Distances were computed using the Kimura 2-parameter model. The numbers at the nodes indicate bootstrap support values >80 for that node. Scale bar represents nucleotide substitutions per site. Black circles indicate the strain from this study. GenBank accession numbers for published sequences are shown in the tree. Full-length sequences obtained from GenBank included 52 HPeV1, 3 HPeV2, 43 HPeV3, 8 HPeV4, 8 HPeV5, 7 HPeV6, 1HPeV7, 2HPeV8, 1 HPeV14, 1 HPeV16, 5 HPeV17, 1 HPeV18 and 1 HPeV19. For clarity, HPeV types have been collapsed. The summary of the HPeV strains used for the phylogenetic analysis is available in the Supplementary Table 2.

The dataset for our phylogenetic analysis of VP1 was conducted with ten HPeV15 VP1 sequences available in GenBank (including three complete and seven partial). All eleven HPeV15 strains clustered together and formed a clade supported by a high (100%) bootstrap value (Fig. 1). HPeV sequences formed monophyletic groups that corresponded to their type designation based on the VP1 region. The highest identity of the study strain was with strain A38 from Ghana (KY931650) (88.2% and 96.9% nt and aa identity, respectively). Overall, strain NIG13194 was more closely related to Ghanaian strains (mean *p*-distance of 0.118 SE ± 0.011) than to strains from Pakistan and Bangladesh (mean *p*-distance of 0.216 SE ± 0.018). Similarly, in terms of aa identity, NIG13194 was also more closely related to African strains (mean *p*-distance of 0.028 SE ± 0.009) than to Asian strains (mean *p*-distance of 0.053 SE ± 0.014). The average *p*-distance in the VP1 region of strain NIG13194 from other HPeV15 strains was 0,172 suggesting great genetic divergence among them. Phylogenetic analysis based on the P1, P2 and P3 coding regions of study strain NIG13194 and complete genome HPeVs strains available in GenBank (n = 134) was conducted to investigate their genetic relationships (Fig. 2). Consistent with the VP1 tree, in the phylogenetic analysis based on the P1 capsid coding region, the NIG13194 isolate clustered with strains of types -3, -17 and -18 (Fig. 2a). No sub-cluster was observed with HPeV15 strains because full-length sequences of the P1 region were not available for published HPeV15 strains. However, in the P2 and P3 region-based trees, strain NIG13194 grouped together with strains of other types sharing the highest similarity with HPeV4 strain TW00032 (83.3%) and HPeV2 strain 51Chzj674 (77.6%), respectively (Fig. 2b,c). These topological inconsistencies between the structural and nonstructural regions are suggestive of recombination during the evolution of HPeV15 or the other HPeV types.

### Recombination analysis

**Figure 3:**
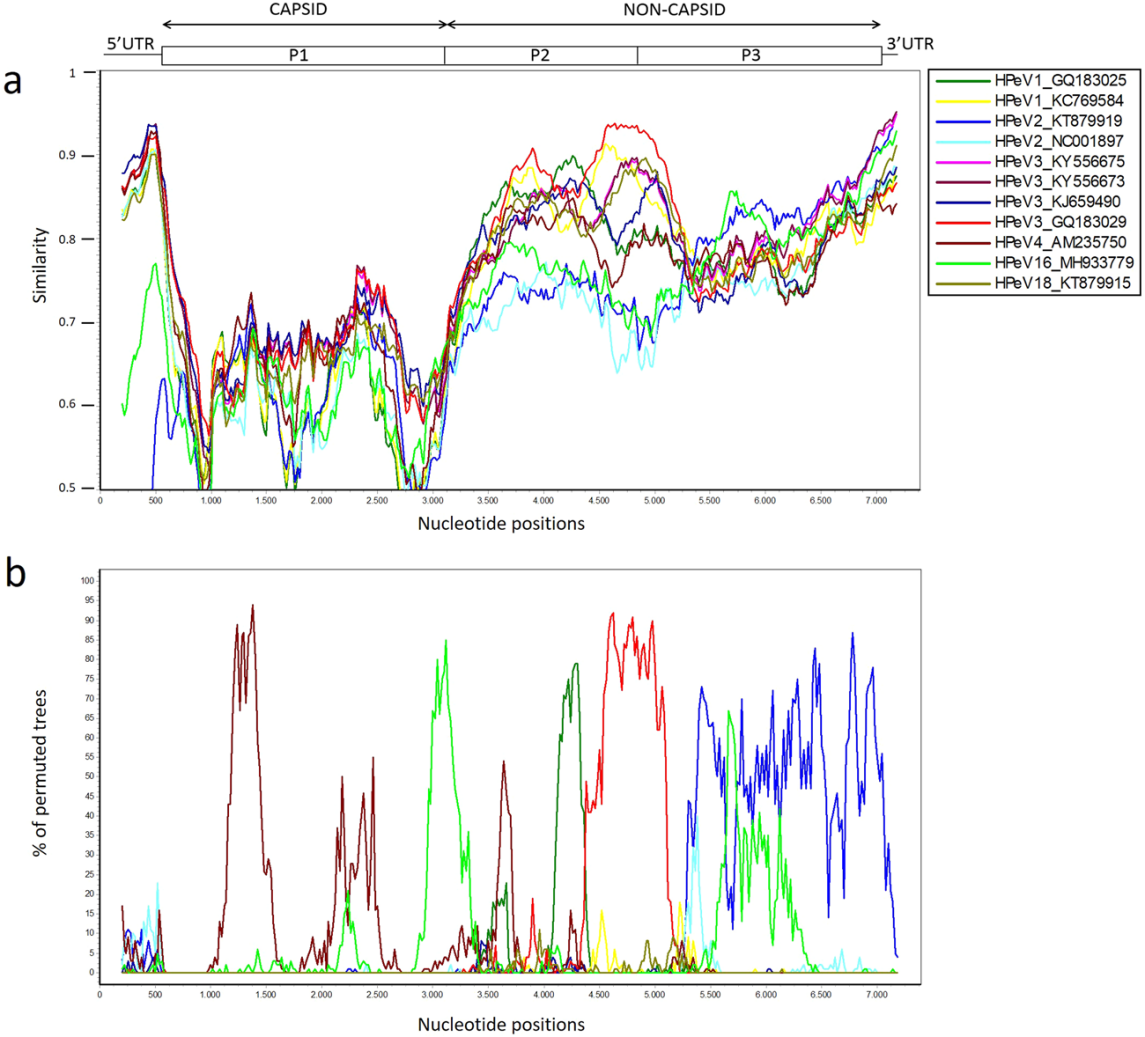
Plot of similarity (**a**) and bootscanning analysis (**b**) of the HPeV15 study strain NIG13194 with closely related strains. The parechovirus genetic organization is shown in the top panel. Analyses were conducted by using SimPlot 3.5.1 (Kimura distance model, window size 400 bp moving in 20 nucleotides steps). NIG13194 was used as a query sequence.

To confirm the existence of recombination events, similarity plot and bootscanning analysis were conducted against sequences closely related to NIG13194. Given that no close relationship was observed between strain NIG13194 and other types based on homologous comparison of different genomic regions with HPeV prototypes (Table 1) or on the phylogenetic analysis in P2 and P3 coding regions (Fig. 2b,c), strains closely related with NIG13194 were screened against the NCBI non-redundant nt database using BLASTn (http://www.ncbi.nlm.nih.gov/). Non-coding regions (5′and 3′UTRs), structural P1 coding region (VP0, VP3 and VP1) and nonstructural P2 (2 A, 2B and 2 C) and P3 (3 A, 3B, 3 C and 3D) regions of NIG13194 were used as queries for BLASTn and sequences with highest homologies and complete genomes were used in the recombination analysis (Supplementary Table 1). A BLASTn search with the complete genome of NIG13194, the complete ORF and the P2 region showed that the highest sequence similarities (81.9%, 81.5% and 87.3%) were shared with an HPeV3 strain (651689) from the Netherlands (Supplementary Table 1). The similarity plot analysis indicate that the genome of strain NIG13194 displays a mosaic-like structure, suggesting that multiple genetic exchanges occurred through recombination events with common ancestors (Fig. 3a). The highest percent support values (>90%) were with different HPeV3 strains at the 5′UTR (HPeV3 strain BJC3174, China 2012), the 3′UTR (HPeV3 strains FEC21 and FEC23, Australia 2013) and the 2 C and 3 A coding regions (HPeV3 strain 651689, Netherlands 2006) which suggests that genetic exchanges might have occurred with type 3 strains. Bootscanning analysis confirmed the existence of recombination events between the genomic sequence of study strain NIG13194 and related viruses (Fig. 3b) and recognized two major potential breakpoints around nucleotide position 3050 in the junction between P1 and the P2 regions and another one located at the 3′-terminus of the 3 A region around nucleotide 5249 (positions referred to alignment).

## Discussion

The present study reports the identification and complete genomic characterization of HPeV15 recovered from a patient in Niger. HPeV15 is a rarely reported HPeV type worldwide. From 2007 to 2010, a limited number of HPeV15 strains were reported from Bangladesh, Pakistan and Ghana^4,9–11^. To the best of our knowledge, HPeV15 has not been reported from elsewhere to date despite continued HPeV surveillance in developed settings such as USA^15,16^ or the Netherlands^17,18^ nor in worldwide HPeV investigations^19–22^. There are two possible reasons for limited worldwide HPeV15 detection: (i) silent transmission due to HPeV underdiagnosis explained by the lack of HPeV routine testing in clinical settings and lack of awareness among clinicians^1^, or (ii) localized geographical circulation of the virus in parts of Africa and Asia. However, in Africa, none of the studies conducted between 2002 and 2016 for HPeV detection in stool samples detected HPeV15 strains, neither in healthy nor in children with gastrointestinal symptoms^23–26^, except for one study from Ghanaian children in 2007-2008^11^. Nevertheless, these studies highlight the high genetic diversity of HPeVs in Africa, which include rare types like HPeV16, HPeV17 or the new type HPeV18^11,23,25,26^. The studies that detected HPeVs in respiratory samples in African countries (Tunisia, Kenya and Gabon) did not perform type identification and therefore it is not possible to know whether type 15 was detected in these samples^27–30^. HPeV typing on respiratory samples would provide valuable information to understand the range of clinical presentations associated with different HPeV types and their genetic diversity. Pairwise and homologous comparison of study strain with VP1 sequences of other HPeV15 strains (African and Asian strains) revealed a high level of genetic diversity between strains. When compared with the HPeV15 prototype, a genetic divergence of more than 20% (79.6%) was observed. Considering the 77% identity demarcation criteria for HPeV types, it is reasonable to conclude that HPeV15 is not a new emerging virus, but one that has been circulating and evolving unrecognized for a long time.

In our study, HPeV15 was identified by using a cell culture system designed for EV diagnostics and a metagenomic approach. This finding differs from previous studies on HPeV15 where the virus was detected without isolation and directly from collected stool samples by HPeV real time reverse transcription PCR and where typing was done by amplifying and sequencing VP1^4,9,11^. We describe the efficient growth of HPeV15 in RD cells, a cell line included as part of the routine diagnostic algorithm implemented for Polio diagnosis. This is in agreement with previous studies describing HPeV types 1 and 4 growth in this cell line^5^. Remarkably, HPeV3, one of the most similar types to our study strain, cannot be cultured in RD cells^5^. Because HPeV has an indistinguishable CPE from EVs in RD cells^31,32^, HPeVs might be misdiagnosed when using conventional culture algorithm designed for EV diagnostics. This calls for implementation of HPeV molecular assays for direct detection of HPeVs in clinical specimens obtained from young children or the use of metagenomics. In this study, untargeted NGS has proved a useful tool when applied retrospectively to screen for coinfecting viruses that are not investigated in routine laboratory work. In recent years, NGS has boosted identification of unsuspected and novel viruses and is becoming the standard for the discovery of viral pathogens^33,34^. It has been successfully used to identify or characterize full-genomes of new HPeVs like a HPeV1 variant^35^, type 4^31^, types 5 and 6^32^, type 7^36^, type 16^26^, type 17^37^, types 18 and 19^38^ or to detect HPeVs in clinical specimens^25,26,39–41^. Thanks to NGS, we were able to detect a mixed infection of HPeV15 and EV-A71 when using this technique to characterize the full genome sequence of EV-A71^13^. Several studies have reported HPeVs in mixed infections. Interestingly, two studies observed that HPeV15-positive cases had a higher chance of confections^10,11^. More studies have observed HPeV coinfections in patients with gastrointestinal or respiratory infections, although the clinical impact of HPeVs in these mixed infections is not clear^20,22,42^.

In our study, HPeV15 has been found in a patient with AFP. HPeVs are increasingly recognized to be associated with viral neurological pediatric disease^2^. Different case reports describe HPeV types 1, 3, 5, 6 and 12 detection in sporadic cases of paralysis in children <3 years of age ^36,40,43–46^. However, we cannot speculate that HPeV15 infection may correlate with AFP since only a few strains have been discovered worldwide and none of them was from AFP cases. In this study, AFP could be explained by the coinfecting EV-A71 (genus *Enterovirus*, family *Picornaviridae*) which has been associated with severe and sometimes fatal neurological diseases, including AFP, affecting mostly infants and children^47^. However, like HPeVs, detection of EVs in feces is not enough evidence to demonstrate the relationship with AFP since asymptomatic long-term excretion of these picornaviruses in stool is common in young children.

Many picornaviruses contain an arginine-glycine-aspartic acid (RGD) motif in the C-terminus of VP1 capsid protein known to play a role in host cell recognition through interactions with cell surface integrins^3^. A functional RGD motif present in HPeV types 1, 2, 4, 5, and 6, while absent in the other HPeV types including type 15, has been associated with infection of the CNS and neonatal sepsis^3,4,45^. In accordance to this, no such motif was detected in the HPeV15 strain found in this study, supporting the use of an alternative non-integrin receptor for entry into host cells although this remains to be determined^1^. It has been suggested that a different cell tropism conferred through the use of a different putative receptor may account for more severe neurological disease symptoms^1,48^. Further clinical studies are required to assess the role of receptor usage and pathogenesis in HPeV infection.

Like for other picornaviruses, one of the evolutionary mechanisms that shape HPeV genomic diversity is the accumulation of mutations, insertions or deletions^49^. When comparing the HPeV15 study strain to the prototype strain, a striking feature was the deletion of nine nt at the 3′ end of the VP1. The capsid protein VP1 is involved in virus binding to cell surface receptors. Whether this deletion in the VP1 protein of the HPeV15 study strain might affect pathogenicity through an altered interaction with host cell receptors warrants further investigation. Genetic recombination is also common process during evolution of HPeVs^48–50^. In agreement with previous studies^38,48,50^, the inconsistent phylogenies across the genome between provide evidence that recombination events have occurred in the evolutionary process of HPeV^49^. Moreover, similarity plot and boot scanning analysis with closely related sequences showed high (>90%) sequence similarity for worldwide distributed HPeV3 strains within the UTRs and the P2/P3 junction, suggesting potential recombination events in these regions. These results correlate with other studies that described the most frequent sites for HPeV recombination in the UTRs or nonstructural region^50,51^. However, despite the high similarity, we cannot infer that any of these HPeV3 strains are the exact recombination donor strain of these regions. Interestingly, the most similar strain in the P2/P3 junction, strain 651689, was identified in stool samples from children aged <5 years in the Netherlands (2006)^52^. This strain does not cluster anymore with other HPeV3 sequences when the nonstructural region is analysed^51^. The HPeV parental strain that recombined with this divergent Dutch strain is unknown, but it is plausible to hypothesize that our study strain and the divergent HPeV3 strain 651689 shared a recent common ancestor. Since these two strains belong to different types, this finding suggests potential intertypic recombination during HPeV15 evolution. Of note, it cannot be excluded recombination with HPeV types 9 to 13 as no whole-genomes sequences are currently available for these types. The fact that the countries of origin of viruses closely related to the HPeV15 strain are geographically far from Niger (China, Australia or The Netherlands, see Supplementary Table 1) and temporally distant from the study strain (Supplementary Table 1), supports the hypothesis that HPeV15 has been circulating globally throughout the years before a recombinant virus emerged.

The scarce number of published HPeV full-genome sequences limits interpretation of results for recombination analysis. More HPeV virus strains and complete genome sequences are needed to better monitor HPeV evolution, divergence and recombination events. This should be complemented with increased surveillance, implementation of molecular detection and typing assays in clinical settings and large-scale epidemiological studies of HPeVs to monitor novel types and further our understanding of their geographic distribution, circulation patterns, genetic diversity, type specific virulence and clinical relevance.

## Materials and Methods

### Ethics statement

The study did not involve human participants or experimentation, but the use of cell culture isolates of viruses recovered from stool samples of patients with AFP collected through routine poliomyelitis surveillance activities at the instigation of the World Health Organization (WHO) for public health purposes. All technical and ethical aspects were approved by WHO and the Ministry of Health of Niger. The protocol for routine AFP surveillance activities framed in the Global Polio Eradication Initiative were approved by the Ethics Committee of the WHO in compliance with applicable National regulations governing the protection of human subjects. The protocol was in accordance with the principles of the Helsinki Declaration. Informed consent was acquired from parents for the use of clinical samples at the time of sample collection.

### Sample collection and virus isolation

According to the WHO guidelines for poliomyelitis surveillance, two stool specimens from all AFP cases under 15 years of age should be collected 24–48 hours apart and within one month of the onset of paralysis^53^. In this study, only one stool sample was collected on March 6^th^ and submitted on March 8^th^, 2013. The sample was collected 8 days after the onset of paralysis (February 26^th^). Following the WHO guidelines for the virological investigation of poliomyelitis^54^, stool samples are processed and inoculated on two continuous cell lines (RD and L20B). For samples showing no cytopathic effect (CPE) after 5 days, a blind passage in the same cell line was done using the supernatant of the inoculated negative cultures and microscopically checked over the next 5 days. Viral cultures positive in RD cells were re-passaged in L20B cells and examined for 5 days to exclude the possibility that they were polioviruses. Only viral cultures producing CPE in RD cells and not in L20B cells were considered to contain NPEVs. Supernatant from RD cells showing a complete CPE was recovered and kept frozen (−20 °C) until typing.

### Metagenomic sequencing

Extraction of RNA was performed as previously described^55^ followed by treatment with Turbo DNase (Ambion) to digest contaminating DNA. Host rRNA were depleted from RNA samples using the NEBNext® rRNA Depletion Kit (New England Biolabs) as described previously^56^. Importantly, samples were processed in a facility where no research on HPeVs has been performed. RNA from selective depletion was used for cDNA synthesis and Illumina library preparation using the Nextera XT kit with dual indexes, and sequenced on a NextSeq. 500 (75 cycles, paired-end reads; Illumina) platform.

### Genome assembly

Raw paired-end files were processed for removal of Illumina adaptor sequences, trimmed and quality-based filtered using Trimmomatic software v0.36^57^. De novo assembly was performed using metaSPAdes with default parameters^58^. Contigs were queried against the ViPR database^59^ retrieved in march 2019 using DIAMOND^60^. In addition to contigs matching EVA-71 (7345 (0.02%) reads mapped to this virus), whose sequence was previously obtained^13^, two large contigs corresponded to HePVs sequences. Using the sequence from the top DIAMOND hits (ADA79690, ANJ01603) as reference, HePVs contigs were assembled into a full-length HePV scaffold with missing sections (gaps). Iterative mapping with the mapper of clc-assembly-cell v5.1.0 (with length fraction set to 0.6) using the total reads on this gapped scaffold allowed to complete the missing sections and obtain a near complete genome (15272 (0.04%) reads mapped to HePV15). A final iteration of the mappings was performed with stringent parameters.

### Phylogenetic analysis

Nt and aa sequence alignment was performed by using ClustalW multiple alignment program within the BioEdit Sequence Alignment Editor package, version 7.0.9.0. Maximum-likelihood (ML) phylogenies of HPeV strains were inferred using IQ-TREE, and branch support was calculated using ultrafast bootstrap approximation with 1000 replicates^61,62^. Prior to the tree reconstruction, the ModelFinder application^63^, as implemented in IQ-TREE, was used to select the best-fitted nt substitution model. Sequence divergence was determined by using MEGA5 to calculate mean pairwise distances within groups.

### Recombination analysis

Similarity plot and bootscanning analysis were performed by using the SimPlot program, version 3.5.1, with a 400-nt window moving in 20-nt steps and using a Kimura two-parameter method with a transitions-transversions ratio of 2 with 1000 resampling.

## Supplementary information

Supplementary information.

## Data Availability

The nt sequence of the complete genome of the HPeV15 strain NIG13194 has been deposited in the GenBank database (Accession No. MN265386). The raw sequence files are available at NCBI under BioProject Accession No. PRJNA564393.
