# Supplementary material for: Identification and molecular characterization of the first complete genome sequence of Human Parechovirus type 15
Source: Sci Rep. 2020 Apr 21;10:6759. doi: 10.1038/s41598-020-63467-w (PMC7174385; doi:10.1038/s41598-020-63467-w)
Supplement: Supplementary file 1 — Supplementary information. [file 41598_2020_63467_MOESM1_ESM.pdf]

Identification and molecular characterization of the first complete genome sequence of Human Parechovirus type 15

Maria Dolores Fernandez-Garcia[1#*], Etienne Simon-Loriere[2#], Ousmane Kebe[1], A. Sakuntabhai[2], Kader Ndiaye[1]

1. Institut Pasteur, Dakar, Senegal
2. Institut Pasteur Paris, France

# Equal contribution

* Corresponding author dolores.fernandez@yahoo.es

Supplementary information

Supplementary Figure 1. Phylogenetic tree of partial VP1 in amino-acids (250 aa in length) including the study strain, global HPeV15 strains and representative global HPeV strains from the GenBank database. The black circle indicates HPeV15 isolate in this study. The maximum likelihood tree was constructed using IQ-TREE using the LG+G4 substitution model, with ultrafast bootstrap. Bootstrap values above 80% are indicated in branch nodes. Scale bars indicate amino acid substitutions per site. The name of each strain includes type designation, GenBank accession number, strain name, country of origin, and year of detection.

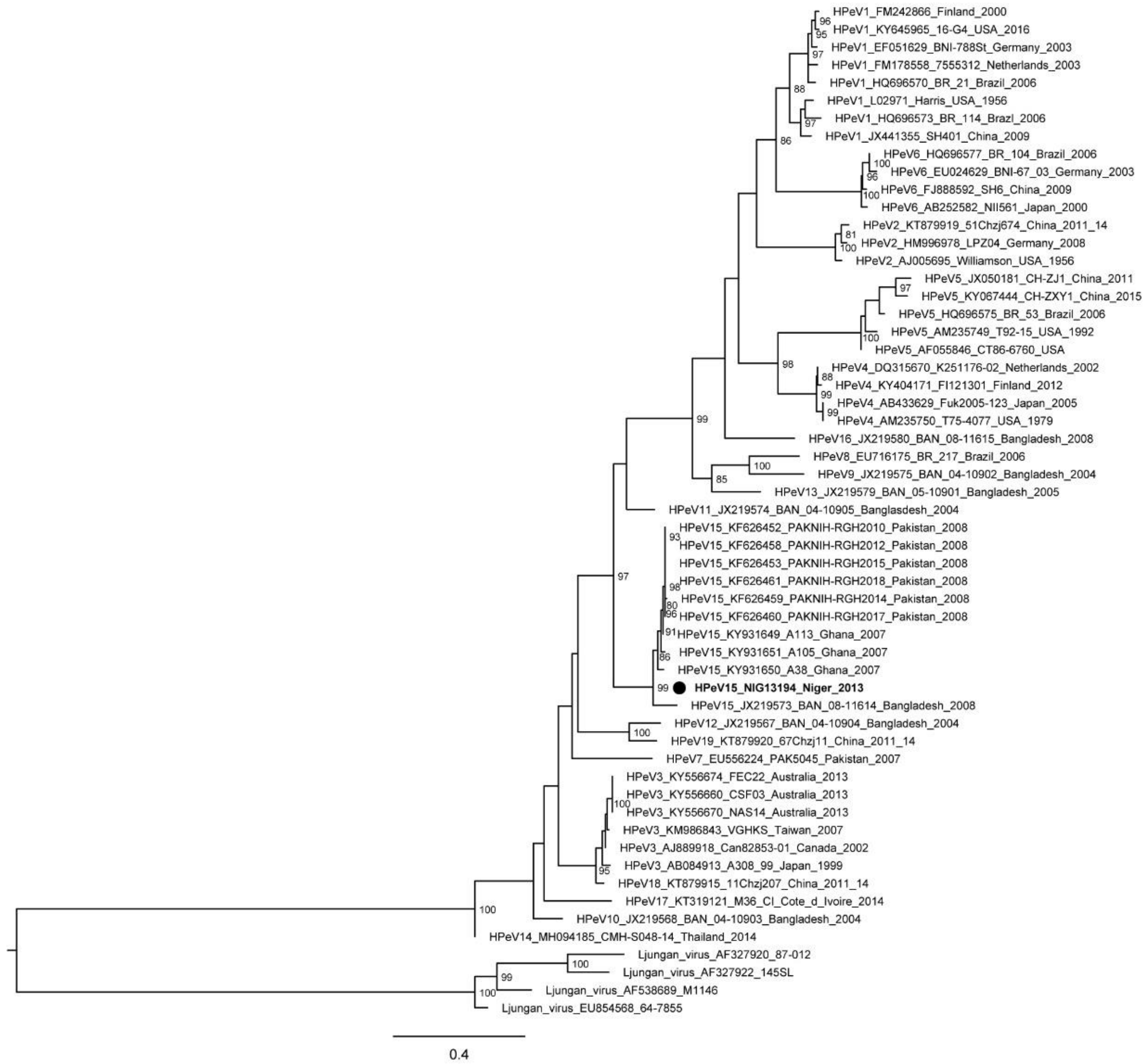
HPeV1_FM242866_Finland_2000
HPeV1_KY645965_16-G4_USA_2016
HPeV1_EF051629_BNI-788St_Germany_2003
HPeV1_FM178558_7555312_Netherlands_2003
HPeV1_HQ696570_BR_21_Brazil_2006
HPeV1_L02971_Harris_USA_1956
HPeV1_HQ696573_BR_114_Brazl_2006
HPeV1_JX441355_SH401_China_2009
HPeV6_HQ696577_BR_104_Brazil_2006
HPeV6_EU024629_BNI-67_03_Germany_2003
HPeV6_FJ888592_SH6_China_2009
HPeV6_AB252582_NII561_Japan_2000
HPeV2_KT879919_51Chzj674_China_2011_14
HPeV2_HM996978_LPZ04_Germany_2008
HPeV2_AJ005695_Williamson_USA_1956
HPeV5_JX050181_CH-ZJ1_China_2011
HPeV5_KY067444_CH-ZXY1_China_2015
HPeV5_HQ696575_BR_53_Brazil_2006
HPeV5_AM235749_T92-15_USA_1992
HPeV5_AF055846_CT86-6760_USA
HPeV4_DQ315670_K251176-02_Netherlands_2002
HPeV4_KY404171_FI121301_Finland_2012
HPeV4_AB433629_Fuk2005-123_Japan_2005
HPeV4_AM235750_T75-4077_USA_1979
HPeV16_JX219580_BAN_08-11615_Bangladesh_2008
HPeV8_EU716175_BR_217_Brazil_2006
HPeV9_JX219575_BAN_04-10902_Bangladesh_2004
HPeV13_JX219579_BAN_05-10901_Bangladesh_2005
HPeV11_JX219574_BAN_04-10905_Banglasdesh_2004
HPeV15_KF626452_PAKNIH-RGH2010_Pakistan_2008
HPeV15_KF626458_PAKNIH-RGH2012_Pakistan_2008
HPeV15_KF626453_PAKNIH-RGH2015_Pakistan_2008
HPeV15_KF626461_PAKNIH-RGH2018_Pakistan_2008
HPeV15_KF626459_PAKNIH-RGH2014_Pakistan_2008
HPeV15_KF626460_PAKNIH-RGH2017_Pakistan_2008
HPeV15_KY931649_A113_Ghana_2007
HPeV15_KY931651_A105_Ghana_2007
HPeV15_KY931650_A38_Ghana_2007
HPeV15_NIG13194_Niger_2013
HPeV15_JX219573_BAN_08-11614_Bangladesh_2008
HPeV12_JX219567_BAN_04-10904_Bangladesh_2004
HPeV19_KT879920_67Chzj11_China_2011_14
HPeV7_EU556224_PAK5045_Pakistan_2007
HPeV3_KY556674_FEC22_Australia_2013
HPeV3_KY556660_CSF03_Australia_2013
HPeV3_KY556670_NAS14_Australia_2013
HPeV3_KM986843_VGHKS_Taiwan_2007
HPeV3_AJ889918_Can82853-01_Canada_2002
HPeV3_AB084913_A308_99_Japan_1999
HPeV18_KT879915_11Chzj207_China_2011_14
HPeV17_KT319121_M36_CI_Cote_d_Ivoire_2014
HPeV10_JX219568_BAN_04-10903_Bangladesh_2004
HPeV14_MH094185_CMH-S048-14_Thailand_2014
Ljungan_virus_AF327920_87-012
Ljungan_virus_AF327922_145SL
Ljungan_virus_AF538689_M1146
Ljungan_virus_EU854568_64-7855
0.4

Supplementary table 1. HPeV strains with the highest similarity in the nucleotide sequences in all the genomic regions of the NIG13194 strain using BLASTn.

Genomic region	Type	Strain	% nucleotide identity	Accession number	Genome	Year of sample collection	Country of origin
**5'UTR**	HPeV3	BJ-C3174	90.3	KJ659490	complete	2012	China
**VP0**	HPeV4	T75-4077	77.1	AM235750	complete	1979	USA
**VP3**	HPeV1	KVP6	75.3	KC769584	complete	2007	Taiwan
**VP1**	HPeV15	GhanaA38	88.5	KY931650	partial	2007	Ghana
**2A**	HPeV18	11Chzj207	79.2	KT879915	complete	2011-14	China
**2B**	HPeV1	K63-94	88.6	GQ183025	complete	1994	Netherlands
**2C**	HPeV3	651689	90.2	GQ183029	complete	2006	Netherlands
**3A**	HPeV3	651689	92	GQ183029	complete	2006	Netherlands
**3B**	HPeV2	Gregory	86.4	AJ005695	complete	--	--
**3C**	HPeV16	CMRHP2	83.5	MH933779	complete	2014	Cameroon
**3D**	HPeV2	51Chzj674	87.7	KT879919	complete	2011-14	China
**3'UTR**	HPeV3	FEC23	96.8	KY556675	complete	2014	Australia
**P1**	HPeV15	GhanaA38	88.1	KY931650	partial	2007	Ghana
**P2**	HPeV3	651689	87.3	GQ183029	complete	2006	Netherlands
**P3**	HPeV3	FEC21	85.3	KY556673	complete	2014	Australia
**ORF**	HPeV3	651689	81.5	GQ183029	complete	2006	Netherlands

Supplementary Table 2. Summary of human parechovirus strains in collapsed groups used for phylogenetic analysis in figure 2. Strain names are indicated by GenBank accession number, isolate code, country of origin and year of isolation. HPeV, Human Parechovirus

Type	Name of strain	Type	Name of strain	Type	Name of strain
HPeV1	L02971_Harris_USA_1956	HPeV1	HQ696574_BR/145_Brazil_2006	HPeV3	KY556670_NAS14_Australia_2013
	EF051629_BNI-788St_Germany_2003		HQ696571_BR/27_Brazil_2006		KY556668_FEC17_Australia_3013
	FM242866_Finland_2000		HQ696572_BR/30_Brazil_2006		KY556661_CSF06_Australia_2013
	KY645965_16-G4_USA_2016		GQ183018_152478_Netherlands_2001		KY556664_CSF07_Australia_2013
	KY645963_16-G10		MG026490_ETH_P28_Ethiopia_2016		KY556666_FEC10_Australia_2013
	MH781721_USA/TN/2016-23032		MG026486_ETH_P5_Ethiopia_2016		KY556663_CSF05_Australia_2013
	KT626007_TW-71594-2010		MG026487_ETH_P6_Ethiopia_2016		KY556662_CSF04_Australia_2013
	GQ183019_252581_Netherlands_2002		GQ183021_550163_Netherlands_2005		KY556660_CSF03_Australia_2013
	JX575746_CAU10NN_South_Korea_2011		GQ183020_450343_Netherlands_2004		KY020128_GL-SA1_Australia_2015
	GQ183025_K63-94_Netherlands_1994		MH933781_CMRHP48_Cameroon_2014		KY556672_FEC20_Australia_2013
	GQ183024_K54-94_Netherlands_1994		KT879927_134Chzj01_China_2011/14		KY556669_NAS16_Australia_2013
	GQ183023_K150-93_Netherlands_1993		KT879923_91Chzj242_China_2011/14		KY556667_FEC12_Australia_2013
	GQ183022_K129-93_Netherlands_1993		KT879917_37Chzj76_China_2011/14		KY556665_CSF08_Australia_2013
	KT879925_112Chzj32_China_2011/14		KT726985_2-B9-TW_Taiwan_2008		KY556659_CSF01_Australia_2013
	KJ659491_BJ-37359_China_2012		MG873158_NS12-N3623_Russia_2012		KM986843_VGHKS_Taiwan_2007
	KT626012_TW-50192_Taiwan_2012		MG873157_NS12-N3567_Russia_2012		AB668030_1356-130823_Japan_2008
	FJ840477_SH1_China_2008		GQ183034_863_Netherlands_2007		AB668033_1448-11832_Japan_2008
	KC769584_KVP6_Taiwan_2007		MG026489_ETH_P16_Ethiopia_2016		AB668032_1443-175790_Japan_2008
	KT626011_TW-01679_Taiwan_2012		GQ183035_452568_Netherlands_2004		AB668031_1357-130823_Japan_2008
	KT626008_TW-71157_Taiwan_2011		KJ152442_MX_Mexico_2009		AB668029_1361K-162589_Japan_2008
	KY460516_TW-00010_Taiwan_2010		KT879926_121Chzj091_China_2011/14		KX068679_VGHKS080217_Taiwan_2013
	KT626006_TW-01319_Taiwan_2010		JX441355_SH401_China_2009		KJ659490_BJ-C3174_China_2012
	KT626005_TW-02680_Taiwan_2008		HQ696573_BR/114_Brazil_2006		JX826607_BONN-2_Germany_2010
	FM178558_7555312_Netherlands_2003		MH933780_CMRHP46_Cameroon_2014		KT626009_TW-03067_Taiwan_2011
	KT879928_146Chzj02_China_2011/14	HPeV2	AJ005695_Williamson_USA_1956		AJ889918_Can82853-01_Canada_2002
	MF371337_CAU91_South_Korea_2015		KT879919_51Chzj674_China_2011/14		GQ183033_K8-94_Netherlands_1994
	KT879930_164Chzj909_China_2011/14		HM996978_LPZ04_Germany_2008		GQ183028_450936_Netherlands_2004
	HQ696570_BR/21_Brazil_2006	HPeV3	KY556673_FEC21_Australia_2013		GQ183027_251360_Netherlands_2002

Type	Name of strain	Type	Name of strain
HPeV3	GQ183032_K20-94_Netherlands_1994	HPeV5	AF055846_CT86-6760_USA
	GQ183030_K11-94_Netherlands_1994		AM235749_T92-15_USA_1992
	GQ183031_K12-94_Netherlands_1994		KT879918_44Chzj84_China_2011/14
	GQ183026_152037_Netherlands_2001	HPeV6	HQ696577_BR/104_Brazil_2006
	AB084913_A308/99_Japan_1999		EU024629_BNI67/03_Germany_2003
	MF371336_CAU70_South_Korea_2015		AB252582_NII561_Japan_2000
	MF371335_CAU48_South_Korea_2015		EU077518_823_Netherlands_2005
	MF371334_CAU14_South_Korea_2015		MG462718_AFW_Australia_2001
	MF371333_CAU11_South_Korea_2015		FJ888592_SH6_China_2009
	JX682576_BONN-1_Germany_2010		KT879921_71Chzj169_China_2011/14
	GQ183029_651689_Netherlands_2006	HPeV7	EU556224_PAK5045_Pakistan_2007
	KY556674_FEC22_Australia_2013	HPeV8	EU716175_BR/217_Brazil_2006
	KY556671_CSF19_Australia_2013		MG571811_V8D_Venezuela_2015
	KY556675_FEC23_Australia_2013	HPeV14	MG571809_V3C_Venezuela_2015
HPeV4	DQ315670_K25117602_Netherlands_2002	HPeV16	MH933779_CMRHP2_Cameroon_2014
	KY404171_FI121301_Finland_2012	HPeV17	KT319121_M36/CI_Cote_d'Ivoire_2014
	KY404170_FI121290_Finland_2012		KT879916_27Chzj76_China_2011/14
	KY404169_FI121236_Finland_2012		KT879924_103Chzj412_China_2011/14
	KY271948_USA/TN/2015-OB2038_USA_2015		KT879922_89Chzj14_China_2011/14
	AB433629_Fuk2005-123_Japan_2005		KT879929_157Chzj058_China_2011/17
	AM235750_T75-4077_USA_1979	HPeV18	KT879915_11Chzj207_China_2011/14
	KT626010_TW-00032_Taiwan_2011	HPeV19	KT879920_67Chzj11_China_2011/14
HPeV5	HQ696575_BR/53_Brazil_2006		
	HQ696576_BR/77_Brazil_2006		
	JX050181_CH-ZJ1_China_2011		
	KY067444_CH-ZXY1_China_2015		
	MG873159_NS12-N3863_Russia_2012		
